# The Role of Unfolded Protein Response in Coronavirus Infection and Its Implications for Drug Design

**DOI:** 10.3389/fmicb.2021.808593

**Published:** 2021-12-24

**Authors:** Mei Xue, Li Feng

**Affiliations:** State Key Laboratory of Veterinary Biotechnology, Harbin Veterinary Research Institute, Chinese Academy of Agricultural Sciences (CAAS), Harbin, China

**Keywords:** coronavirus, endoplasmic reticulum stress, host innate immunity, drug targets, unfolded protein response (UPR)

## Abstract

Coronavirus is an important pathogen with a wide spectrum of infection and potential threats to humans and animals. Its replication occurs in the cytoplasm and is closely related to the endoplasmic reticulum (ER). Studies reported that coronavirus infection causes ER stress, and cells simultaneously initiate unfolded protein response (UPR) to alleviate the disturbance of ER homeostasis. Activation of the three branches of UPR (PERK, IRE1, and ATF6) modulates various signaling pathways, such as innate immune response, microRNA, autophagy, and apoptosis. Therefore, a comprehensive understanding of the relationship between coronavirus and ER stress is helpful to understand the replication and pathogenesis of coronavirus. This paper summarizes the current knowledge of the complex interplay between coronavirus and UPR branches, focuses on the effect of ER stress on coronavirus replication and coronavirus resistance to host innate immunity, and summarizes possible drug targets to regulate the impact of coronavirus infection.

## Introduction

Coronaviruses (order Nidovirales, family Coronaviridae, subfamily Coronavirinae) are a family of enveloped viruses with positive sense, non-segmented, single-stranded RNA genomes. According to the antigenicity relationship and subsequent sequence comparison of the whole virus genome, the *coronavirinae* is classified into four genera: the α-coronavirus, β-coronavirus, γ-coronavirus, and δ-coronavirus (Masters, 2006). Coronavirus infection has a wide spectrum, which causes lethal respiratory infections in humans, diarrhea in pigs and cattle, and bronchitis in poultry. Transmissible gastroenteritis virus (TGEV) and porcine epidemic diarrhea virus (PEDV), members of the α-coronavirus family (Masters, 2006), are the cause of economically important swine disease. PEDV and TGEV infect and destroy villous epithelial cells of the jejunum and ileum, resulting in lethal watery diarrhea and piglet dehydration (Van Nieuwstadt et al., 1989; Cruz et al., 2011). The two human α-coronavirus (HCoV-229E and HCoV-NL63) cause mild symptoms mainly restricted to the upper respiratory tract (Shaban et al., 2021). Human coronaviruses (SARS-CoV, SARS-CoV-2 and MERS-CoV) are a member of β-coronavirus. The emergence of SARS-CoV in 2003 and the SARS-CoV-2 still epidemic worldwide pose a great threat to human life (Ksiazek et al., 2003; Zarandi et al., 2021). γ-coronavirus mainly infects avian hosts. For example, avian infectious bronchitis virus (IBV) is an acute, highly contagious respiratory infectious disease in chickens. Most strains can cause specific trachea lesions, but some can cause kidney and reproductive tract lesions. The infection of laying hens usually results in a decrease in egg production and egg quality. The disease is widely prevalent worldwide and is an important epidemic disease in the chicken industry (Cavanagh, 2007). Porcine deltacoronavirus (PDCoV) is an emerging porcine intestinal pathogenic coronavirus belonging to the δ-coronavirus family, which caused diarrhea and vomiting in piglets with an incidence rate and mortality rate as high as 50–100% (Zhang et al., 2019).

**FIGURE 1:**
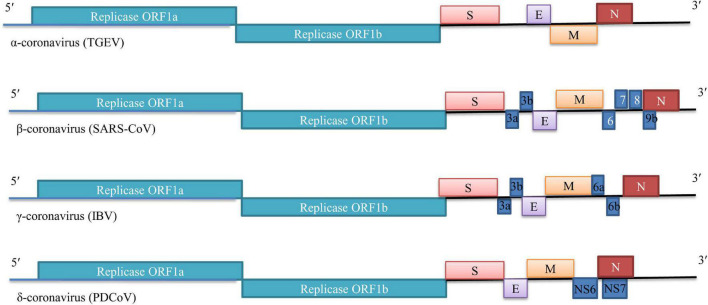
Genome organization of selected members of genus Coronavirinae (TGEV, SARS-CoV, IBV and PDCoV).

Coronaviruses are enveloped positive-sense RNA viruses that range in size from 26.4 to 31.7 kb (Hidalgo et al., 2021). The 5′-proximal two-thirds (∼20–22 kb) of the genome consists of the two largest ORFs (ORF1a and ORF1b) that encode for the non-structural proteins (nsps). The 3′ one-third of the genome encodes the structural proteins-the highly glycosylated spike (S) protein, envelope (E), membrane (M), nucleocapsid (N), as well as several accessory proteins generally non-essential for replication in tissue culture but capable of inhibiting immune responses and enhancing pathogenesis (Figure 1). Infection begins when coronavirus S protein attaches to its complementary host receptor, allowing the virus to enter the host cells through endocytosis or direct fusion of the viral envelope with the cell membrane (Artika et al., 2020). After successfully enters the cells, coronavirus induces profound remodeling of the intracellular membrane network to generate double-membrane vesicles (DMVs). The viral genome RNA is translated to synthesize the viral proteins which are necessary for subsequent RNA replication and transcription. The structural proteins (S, M, and E) are synthesized, inserted, and folded in the ER and transported to the ER-Golgi apparatus intermediate compartment (ERGIC), which is the location of the coronavirus particle assembly, whereas N proteins are translated in the cytoplasm. Following assembly, the progeny virions accumulate in smooth-walled vesicles are transported to the cell surface, and ultimately fuse with the plasma membrane to release the mature virus particle (Artika et al., 2020).

Endoplasmic reticulum with normal function is essential for protein synthesis, folding, modification, and transport (Ron and Walter, 2007; Fung et al., 2014a; Hetz and Papa, 2017). Disturbances in the structure and function of the ER with the accumulation of unfolded/misfolded proteins lead to ER stress. The three major ER stress sensors [protein kinase R-like ER kinase (PERK), inositol-requiring enzyme 1 (IRE1), and activating transcription factor 6 (ATF6)] are activated by accumulated unfolded/misfolded proteins in the ER and initiated the unfolded protein response (UPR) (Credle et al., 2005; Ron and Walter, 2007; Hetz and Papa, 2017). The initiation marker of UPR is the expression of ER chaperone molecules, such as immunoglobulin heavy chain binding protein (BiP/GRP78) and glucose regulatory protein 94 (GRP94). UPR maintains ER homeostasis by turning off the translation of ER proteins and increasing their folding ability. However, UPR can induce apoptosis and cytokine production under sustained ER stress (Ron and Walter, 2007).

Coronavirus genome replication occurs in the cytoplasm, and the synthesis and translation of viral proteins are closely related to ER and its transducers. It is well documented that coronavirus replication causes ER stress and induces UPR in the infected cells. Coronaviruses, such as SARS-CoV-2, SARS-CoV, mouse hepatitis virus (MHV), IBV, TGEV, and PEDV, can all induce significant ER stress following infection (Versteeg et al., 2007; Krahling et al., 2009; DeDiego et al., 2011; Liao et al., 2013; Xue et al., 2018; Echavarria-Consuegra et al., 2021). GRP78 and GRP94 are overexpressed in SARS-CoV infected cells. Some SARS-CoV proteins have been shown to induce ER stress responses, such as spike protein, ORF3a, ORF6, ORF7a, ORF8ab, or ORF8b proteins (Chan et al., 2006; Ye et al., 2008; Minakshi et al., 2009; Sung et al., 2009; Shi et al., 2019). It is recognized that protein processing after synthesis is an important step in gene expression, and protein misfolding plays an important role in coronavirus infection. In the following chapters, we will summarize the latest knowledge on the signaling mechanism of coronavirus induced UPR and the regulation of UPR on coronavirus infection. The significance of the UPR in host innate immune response and possible drug targets will also be discussed.

## Regulation of Coronavirus Replication by Unfolded Protein Response

All three branches of the UPR were induced in MHV, SARS-CoV-2, PEDV, and TGEV infected cells (Versteeg et al., 2007; Xue et al., 2018; Chen et al., 2021; Echavarria-Consuegra et al., 2021). Pharmacological inhibition of the UPR greatly regulated coronavirus replication, revealing the importance of this pathway for successful coronavirus replication.

### The PERK Signaling

**FIGURE 2:**
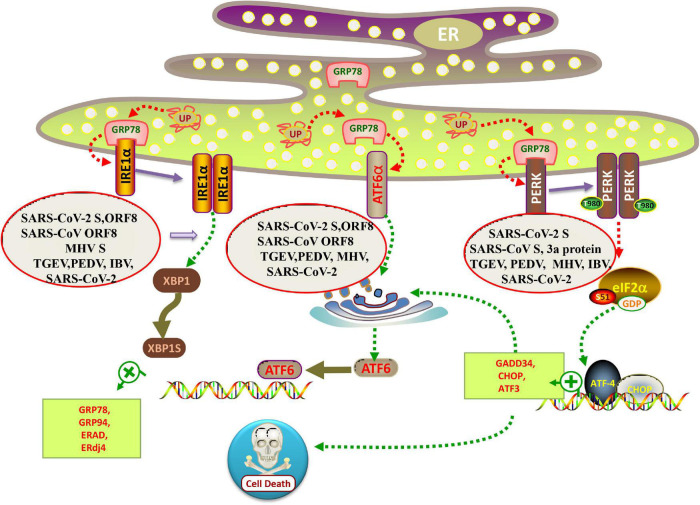
Schematic diagram of coronavirus infection modulating the UPR arms. When misfolded proteins accumulate in the lumen of ER, chaperone binding immunoglobulin (BiP/GRP78) is separated from the lumen domains of the three ER sensors, allowing PERK and IRE1 to form a homodimer and activate the transport of ATF6 to the Golgi. All three branches of the UPR were induced in SARS-CoV-2, MHV, PEDV and TGEV infected cells. Individual over-expression of SARS-CoV and SARS-CoV-2 ORF8 protein is sufficient to induce the IRE1 and ATF6 pathway of UPR. While SARS-CoV-2 S protein activates all three branches of the UPR, SARS-CoV S protein only induces PERK pathway.

PERK belongs to the protein kinase family of the eukaryotic protein translation initiation complex. In the normal state, PERK binds to GRP78. Under conditions of ER stress, PERK dissociates from GRP78, phosphorylates itself, and forms a dimer. Activated PERK can specifically phosphorylate eukaryotic translation initiation factor 2α (eIF2α) at serine 51 (Figure 2). Studies based on transient transfection of SARS-CoV proteins have also implicated the involvement of the PERK branch of UPR (Chan et al., 2006; Krahling et al., 2009; DeDiego et al., 2011). For example, Chan et al. (2006) have shown that over-expression of SARS-CoV spike protein induces upregulation of the ER chaperones GRP78 and 94 through PERK activation. Later, the UPR activating domain of SARS-CoV S protein is mapped to the central region (amino acids 201–400) of the S1 subunit and seems to function independently of N-linked glycosylation (Siu et al., 2014). Interestingly, the accessory protein 3a of SARS-CoV, a small multipass transmembrane protein, also activates the ATF4 and Chop promoter activities and thus may potentially also activate the PERK branch of UPR (Minakshi et al., 2009). MHV infection leads to activation of PERK-eIF2α-ATF4 branch (Figure 2; Echavarria-Consuegra et al., 2021).

Virus replication is closely dependent on the host cellular protein synthetic machinery to produce viral proteins and particles. Several lines of evidence have indicated a link between viral replication and the PERK pathway (Pavio et al., 2003; Datan et al., 2016; Landeras-Bueno et al., 2016; Zhou et al., 2016). Xue et al. (2018) demonstrated that infection with the α-coronavirus TGEV induced ER stress and triggered the three branches of UPR *in vitro* and *in vivo*. Treatment with thapsigargin (Tg) or tunicamycin (Tu) (the ER stress activator) significantly inhibits TGEV replication, indicating that ER stress negatively regulated TGEV replication. Further studies showed that although TGEV infection activated all three ATF6, IRE1, and PERK pathways, their effects on viral replication were different: PERK-eIF2α axis inhibits TGEV replication, IRE1 pathway contributes to virus replication, while ATF6 pathway does not affect TGEV replication. In addition, they demonstrated that phosphorylated eIF2α induced overall attenuation of protein translation, thereby down-regulating TGEV replication (Xue et al., 2018).

Intriguingly, using a recombinant TGEV virus lacking the accessory gene 7 (rTGEV-Δ7), Cruz et al. (2013) have demonstrated that the protein 7 of TGEV physically interacts with PP1 and promotes eIF2α dephosphorylation. Compared with the wild-type virus, cells infected with rTGEV-Δ7 have a much higher phosphorylated eIF2α, resulting in significant translation attenuation and drastic induction of GADD34 (a component of the PP1 complex responsible for the eIF2α dephosphorylation) (Cruz et al., 2013). In cells infected with PEDV, PERK is activated, as seen by an increase in autophosphorylation of PERK during virus infection. Notably, phosphorylation of PERK is dependent on the production of viral protein synthesis. The knockdown of PERK increased virus loads in the cells, consistent with the ER stress inhibitor, 4-phenylbutyrate (4-PBA) treatment (Wang et al., 2014).

In terms of γ-coronavirus, IBV infection activates the PERK-eIF2α-ATF4 and PKR-eIF2α-ATF4 pathways, resulting in the induction of ER stress-mediated proapoptotic pathways (Liao et al., 2013). Interestingly, IBV replication is not significantly affected by knockdown of PERK, indicating that similar to SARS-CoV, IBV is not sensitive to the antiviral activities of PERK *in vitro* (Liao et al., 2013). Thus, the effect of the PERK branch on virus replication is limited to the specific types of coronaviruses.

### The IRE1 Signaling

Mammalian IRE1 has two isoforms-IRE1α and IRE1β. IRE1α is a transmembrane protein with dual enzymatic activities, consisting of an ER luminal amino-terminal domain and a serine/threonine kinase domain plus a carboxyl-terminal ribonuclease (RNase) domain located on the cytosolic side of the protein. Under ER stress, IRE1 becomes an active kinase and autophosphorylates themselves. Splices of the 26-nucleotide intron lead to a frame-shift transcript, which encodes the spliced XBP1 protein (XBP1s) (Yoshida et al., 2001). XBP1s is a potent bZIP transcription factor that induces the expression of genes harboring the UPR element (UPRE) or the ER stress response element (ERSE) in the promoter sequences (Shuda et al., 2003). XBP1s regulate genes involved in protein entry into ER, folding, glycosylation, ER-associated degradation (ERAD), lipid biogenesis, and vesicular trafficking to counteract ER stress (Glimcher, 2010). The expression of at least two genes, the ER DNA J domain-containing protein 4 (ERdj4) and the protein kinase inhibitor of 58 kDa (p58IPK), are specifically induced by XBP1s, but not other UPR transcription factors (Lee et al., 2003).

The IRE1/XBP1 branch is involved in many viral infections. Herpes simplex virus 1 (HSV-1) can avoid cellular responses that are likely detrimental to viral replication by UL41 suppressing the IRE1/XBP1 pathway *via* its RNase activity (Zhang et al., 2017; Zhu and Zheng, 2020). Hepatitis C virus suppresses the IRE1/XBP1 pathway, and influenza A virus induces the IRE1/XBP1 pathway (Tardif et al., 2004; Hassan et al., 2012). For coronavirus, MHV S protein activates the IRE1α-XBP1 pathway (Figure 2; Versteeg et al., 2007). Neither infection with SARS-CoV nor overexpression of SARS-CoV S protein induces XBP1 mRNA splicing (Versteeg et al., 2007; DeDiego et al., 2011). Contrary to SARS-CoV, the expression of the SARS-CoV-2 S protein is sufficient to induce all three major signaling pathways of the UPR (Echavarria-Consuegra et al., 2021). IBV induced ER stress in infected cells and activated the IRE1α-XBP1 pathway at a late stage of infection. Both the kinase and RNase domains of IRE1 are necessary to protect infected cells from IBV induced apoptosis. IRE1 appears to convert it from a proapoptotic unspliced form to an antiapoptotic spliced form (Fung et al., 2014b). Previously, they demonstrated that the PERK branch of the UPR is activated at the early stage of IBV infection (Liao et al., 2013), leading to phosphorylation of eIF2α and upregulation of the Chop/GADD153 (C/EBP-homologous protein or growth arrest and DNA damage-inducible protein 153), which promotes IBV-induced apoptosis by suppressing the prosurvival extracellular signal-related kinase (ERK) (Liao et al., 2013). It is generally believed that when ER stress occurs, PERK is first activated, followed by ATF6 and IRE1 (Ron and Walter, 2007). It is interesting to consider the temporal control of UPR activation and its implication in coronavirus infection. From the host perspective, early activation of the PERK pathway and eIF2α phosphorylation induces translation attenuation, an effective antiviral defense mechanism. The induction of apoptosis through the eIF2α-ATF4-Chop pathway may also restrict virus replication. On the other hand, activation of IRE1 at the late stage of infection promotes the survival of infected cells, allowing more virions to be assembled and released before the infected cells succumb to apoptotic cell death.

In addition to the direct modulation of gene transcription, IRE1 cleaves and leads to the selective degradation of a small set of host microRNA (miRNA). SARS-CoV-2 and other human CoVs regulate host miRNAs to increase ER or ER membrane folding ability advantageously, block UPR related translation attenuation, inflammatory response, and apoptosis, and protect the virus from the influence of the immune system. For example, COVID-19-mediated reduction of host miR-34a-3p and miR-495-5p levels can increase the expression of XBP1s and BiP by increasing the folding ability of ER (Bartoszewski et al., 2020). The IRE1 pathway activated by TGEV infection can downregulate the host miR-30a-5p abundance, while miR-30a-5p targets the 3′UTR region of SOCS1 and SOCS3. The down-regulated expression of miR-30a-5p makes SOCS1 and SOCS3 negatively feedback regulate IFN antiviral signaling (Ma et al., 2018).

### The ATF6 Signaling

ATF6 resides in the ER membrane with a cytosolic amino-terminal domain and an ER luminal carboxyl-terminal domain. As a result of its activation, the amino-terminal domain of ATF6 is released by proteolysis. This portion of ATF6 translocates to the nucleus, where it cooperates with other proteins to form a complex that induces the expression of genes coding for chaperones or folding enzymes. A study of SARS-CoV has found one of the accessory proteins (8ab protein) directly binding to the ATF6 lumen domain, suggesting 8ab protein might facilitate protein folding and processing by modulating UPR (Sung et al., 2009). When SARS-CoV-2 ORF8 was transfected, IRE1α-XBP1 and ATF6 were the main pathways induced (Echavarria-Consuegra et al., 2021). In MHV infected cells, ATF6 was processed to its active form at 7 h after infection, but both full-length ATF6 and active ATF6 disappeared at the later stage of infection (Echavarria-Consuegra et al., 2021). In TGEV-infected cells, ATF6 was activated, but no significant difference in TGEV replication was found after ATF6 knockdown in ST and IPEC-J2 cells by siRNA compared with that of control siRNA. The results indicate that activation of the ATF6 branches is not responsible for suppressing TGEV replication by UPR (Xue et al., 2018).

## Endoplasmic Reticulum Stress on Innate Immune Response

**FIGURE 3:**
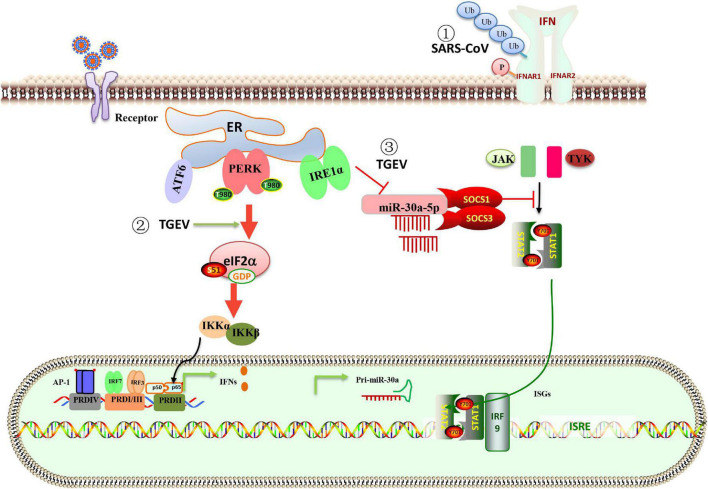
Schematic representation of the interaction between ER stress, UPR, and innate immune system in response to coronavirus infection. (1) The PERK branch of UPR elicited by SARS-CoV 3a protein leads to ubiquitination, phosphorylation, and lysosomal degradation of IFNAR1 and inhibits IFN-I signaling. (2) TGEV infection activates the PERK-eIF2α pathway, the phosphorylation of eIF2α attenuates global protein synthesis, which decreases the levels of cytoplasm IκBα and leads to reduced inhibition of IκBα on NF-κB, promoting IFN-I production. (3) The IRE1 axis reduced host miR-30a-5p abundance, resulting in increased negative feedback regulators of JAK-STAT signaling, SOCS1 and SOCS3. High expression of SOCS1 or SOCS3 disrupted the antiviral effect of IFN-I. The data explain that TGEV evades the IFN-I antiviral response despite the intense induction of endogenous IFN-I.

In the innate immunity of the host, many cytokines are produced to fight the virus. Type I interferon (IFN-I) is the main antiviral molecule, but coronavirus has evolved a variety of strategies, including UPR, to combat IFN-I response during infection. MERS-CoV protein 4a is the first coronavirus protein identified as an antagonist of the dsRNA-dependent, PKR-mediated stress response and IFN-α/β pathways (Rabouw et al., 2016). SARS-CoV activates the PERK pathway of UPR, followed by eIF2α. The activation of the PERK pathway regulates innate immunity by inhibiting the IFN-I signaling. Mechanically, the 3a protein-induced serine phosphorylation and ubiquitination of the IFN alpha-receptor subunit 1 (IFNAR1) leading to lysosomal degradation of IFNAR1 (Figure 3; Minakshi et al., 2009). After TGEV infection, in addition to the classical signaling pathway (Ding et al., 2017), NF-kB can be activated by PERK-eIF2α to induce IFN-I production (Xue et al., 2018). As a member of the α-coronavirus family, PEDV inhibited IFN production, while TGEV induced high levels of IFN production. Surprisingly, the adequate IFN transcription could not suppress viral replication (Zhu et al., 2017). Ma et al. (2018) found a new strategy for TGEV to evade the IFN-I antiviral response by using the IRE1-miR-30a-5p/SOCS1/3 axis. The IRE1 axis reduced host miR-30a-5p abundance, resulting in increased negative feedback regulators of JAK-STAT signaling, SOCS1, and SOCS3. High expression of SOCS1 or 3 disrupted the antiviral effect of IFN-I (Figure 3; Ma et al., 2018). The data explain that TGEV evades the IFN-I antiviral response despite the intense induction of endogenous IFN-I.

The study of IBV found that the eIF2α phosphorylation leads to IκBα decrease, thereby activating NF-κB and promoting cytokine production. Activating the IRE1α-XBP1 signaling pathway is necessary for the production of IL-8 in IBV infected cells (Zhu et al., 2021). IRE1 kinase activity rather than RNase activity is involved in ER stress-mediated NF-κB activation. Therefore, UPR plays an important role in regulating IFN response and innate immunity of coronavirus-infected cells.

## The Unfolded Protein Response-Dependent Molecular Mechanisms as a Therapeutic Target for Coronavirus

Recent data suggest that the disturbance of UPR signal branches may play a role in developing and progressing various human diseases (Banerjee et al., 2020; Rozpedek-Kaminska et al., 2020). The ER stress inducer thapsigargin efficiently inhibits coronavirus (HCoV-229E, MERS-CoV, SARS-CoV-2) replication in different cell types, including primary differentiated human bronchial epithelial cells (Shaban et al., 2021). Thapsigargin exerts a profound antiviral effect on TGEV in two different cell types, including a non-transformed cell line originating from porcine jejunum epithelium (IPEC-J2) (Xue et al., 2018). This compound or its derivatives with better specificity, pharmacokinetics, and safety may be an interesting drug candidate suitable for alleviating the consequences of the potential CoV epidemic in the future. Sigma-1 receptor (Sig-1R) is a ligand-operated ER binding chaperone as an upstream regulator of ER stress and is, therefore, a candidate host protein for host-based re-utilization in patients with COVID-19 (Vela, 2020). Both andrographolide and melatonin provide useful adjuvant therapy for COVID-19 by impacting the UPR signaling pathways by inhibiting the ER stress transducers (Banerjee et al., 2020; Sureda et al., 2020). There is also evidence that 2-Deoxy-D-glucose (2-DG), an ER stress inducer, inhibits PEDV infection *via* altering viral protein translation during the early stage of virus infection and depressing the virus assembly (Wang et al., 2014). Coronavirus induces profound remodeling of primarily ER-derived membranes and triggers the formation of double-membrane vesicles (DMVs) for viral genome replication. Researchers determined the antiviral effect of a small complex inhibitor called K22 [(z)-N-(3-(4-(4-bromophenyl)-4-hydroxypiperidine-1-yl)-3-oxo-1-phenylpropyl-1-ene-2-yl) benzamide] in primary human epithelial cell cultures (Lundin et al., 2014). K22 exerts strong anti-coronavirus activity in the early stage of infection by preventing the formation of DMVs, leading to almost complete inhibition of RNA synthesis, including SARS-CoV and MERS-CoV (Lundin et al., 2014; Sureda et al., 2020). These data suggest that pharmacological regulation of the UPR signaling pathway may significantly inhibit coronavirus replication (Table 1). Therefore, targeting the ER stress-dependent UPR signaling branches may help to develop a new anti-coronavirus treatment strategy.

**TABLE 1:** A list of drugs that modulate ER stress and their anti-coronavirus activities.

Compound	Biological activity	Antiviral activity	
		Virus	References
Thapsigargin	ER stress inducer	HCoV-229E, MERS-CoV, SARS-CoV-2, TGEV	Xue et al., 2018; Shaban et al., 2021
Sig-1R	Chaperone protein in ER	SARS-CoV-2	Vela, 2020
Andrographolide	A NF-κB inhibitor	SARS-CoV-2	Banerjee et al., 2020; Sureda et al., 2020
Melatonin	ATF6 inhibitor	SARS-CoV-2	Banerjee et al., 2020; Sureda et al., 2020
2-DG	Inhibiting glycolysis via its actions on hexokinase	PEDV	Wang et al., 2014
GSK2606414	PERK inhibitor	SARS-CoV-2	Sonkar et al., 2021
Salubrinal	Selective inhibitor of eIF2α dephosphorylation	TGEV	Xue et al., 2018
ISRIB	PERK inhibitor	MHV	Echavarria-Consuegra et al., 2021
4μ8C	IRE1α inhibitor	TGEV	Xue et al., 2018
STF-083010	Inhibits IRE1 endonuclease activity without affecting its kinase activity	SARS-CoV-2, MHV	Echavarria-Consuegra et al., 2021
AEBSF	ATF6 inhibitor	SARS-CoV-2, MHV	Echavarria-Consuegra et al., 2021

Small molecules targeting the PERK branch include GSK2606414, Salubrinal, GSK2656157, ISRIB, Guanabenz, Sepin1, Trazodone Hydrochloride, and Dibenzoylmethane, LDN-0060609, Oxyresveratrol, β-Asarone, and Gastrodia Elata Derivatives (Rozpedek-Kaminska et al., 2020). GSK2606414 is the first-generation and highly selective PERK inhibitor characterized by 30 nm IC50 (the half-maximal inhibitory concentration) and good cell efficacy *in vitro* and *in vivo*. Its selectivity is > 385-fold over other eIF2α kinases. Recently, 12 inhibitors with high binding energies were used to treat COVID-19. GSK2606414 has shown a strong binding affinity with SARS-CoV-2, but it was excluded from the study since its ability to be carcinogenic (Sonkar et al., 2021). GSK2606414 treatment of MHV-infected cells revealed a relief of translation inhibition (Echavarria-Consuegra et al., 2021). In another project, researchers have shown that the silencing of PERK by GSK2606414 considerably elevated TGEV replication in the cells. In addition, the increase in virus loads was correlated with the inhibition efficacy of p-PERK and p-eIF2α (Xue et al., 2018). Salubrinal selectively inhibited eIF2α dephosphorylation and evoked an increased level of p-eIF2α. Treatment of cells with salubrinal demonstrated the opposite effect to previously tested PERK inhibitors and caused a dose-dependent decrease in TGEV replication (Xue et al., 2018). When 2 μM ISRIB was used to treat MHV infected cells, it was found that the transcription of *Chop* decreased and the virus titers reduced by ∼sixfold (Echavarria-Consuegra et al., 2021). Thus, the latest data suggest that using small-molecule inhibitors of PERK-dependent UPR signaling pathways may help develop a novel and pioneering therapeutic strategy against coronavirus.

Small molecule inhibitors that selectively block IRE1α-XBP1 activation have been identified. Two major sites have been explored as targets for IRE1α inhibitors: the ATP binding site of the kinase domain and the catalytic core of the RNase domain. “Compound 3” and APY29 are both IRE1α kinase inhibitors which are reported to inhibit IRE1α-XBP1 by directly interfering with ATP binding in the IRE1α kinase domain (Wang et al., 2012). Small molecules targeting the RNase domain include 4μ8C, STF-083010, salicylaldehydes, toyocamycin, MKC-3946, and hydroxyl-aryl-aldehydes (HAA) (Cross et al., 2012; Jiang et al., 2015). Targeting the RNase activity of IRE1 could potentially be an ideal approach to modulate COVID-19 infection and pathogenesis *via* modulation of the secretome of macrophages. Inhibition of IRE1α RNase activity with small molecule 4μ8c suppressed TGEV replication (Ma et al., 2018). These data suggest that IRE1α-targeting might be a new strategy to cope with coronavirus infection.

AEBSF is a serine protease inhibitor, preventing ER stress-induced ATF6 cleavage, thereby inhibiting the transcriptional induction of ATF6 target genes (Chan et al., 2006). *Calreticulin* and *Grp94* transcription were significantly reduced in AEBSF treated MHV infected cells (Echavarria-Consuegra et al., 2021).

The combination of UPR inhibitors has a cumulative effect on virus release. Cells incubated with STF-083010 (targeting the IRE1α pathway) lead to ∼twofold reductions in MHV titers, while cells treated with STF-083010 and AEBSF (targeting the IRE1α and the ATF6 pathways) reduced virus titer ranged to ∼40- and ∼100-fold. Reductions in virus titer were observed in SARS-CoV-2 infected cells, and the reductions were generally much greater than those seen for MHV, with STF-083010/AEBSF combinations reducing SARS-CoV-2 titers to below the limit of detection (Echavarria-Consuegra et al., 2021).

## Conclusion

Coronavirus has posed a great threat to humans and animals. Despite decades of elegant investigations, the interactions between the host and coronaviruses during infection remain poorly understood. Recent studies have demonstrated that coronavirus infection induces ER stress in infected cells and activates the UPR. Notably, previous studies on coronavirus-induced UPR have been mainly focusing on individual branches of the UPR. It is important to note that the three branches of UPR are not functionally independent but rather operate as an integrated signaling network. The complex of GRP78 and three sensors, PERK, ATF6, and IRE1, regulated by viruses is probably evolved to either regulate the pathological process or optimize viral replication. UPR inhibitors may have dual therapeutic effects, which help reduce viral load and diminish the pathophysiology associated with coronavirus. Further investigation on the molecular interaction between viruses, ER stress, and innate immunity may yield important information to clarify coronavirus pathogenesis. The crosstalk between ER stress and antiviral activities suggests that novel therapeutic targets may have potential utility against coronavirus.

## Author Contributions

MX conceived and drafted the manuscript. LF conceived and revised the manuscript. All authors read and approved the final manuscript for publication.

## Conflict of Interest

The authors declare that the research was conducted in the absence of any commercial or financial relationships that could be construed as a potential conflict of interest.

## Publisher’s Note

All claims expressed in this article are solely those of the authors and do not necessarily represent those of their affiliated organizations, or those of the publisher, the editors and the reviewers. Any product that may be evaluated in this article, or claim that may be made by its manufacturer, is not guaranteed or endorsed by the publisher.

## References

[B1] Artika I. M. Dewantari A. K. Wiyatno A. (2020). Molecular biology of coronaviruses: current knowledge. *Heliyon* 6:e04743. 10.1016/j.heliyon.2020.e04743 32835122 PMC7430346

[B2] Banerjee A. Czinn S. J. Reiter R. J. Blanchard T. G. (2020). Crosstalk between endoplasmic reticulum stress and anti-viral activities: a novel therapeutic target for COVID-19. *Life Sci.* 255:117842. 10.1016/j.lfs.2020.117842 32454157 PMC7245231

[B3] Bartoszewski R. Dabrowski M. Jakiela B. Matalon S. Harrod K. S. Sanak M. (2020). SARS-CoV-2 may regulate cellular responses through depletion of specific host miRNAs. *Am. J. Physiol. Lung. Cell Mol. Physiol.* 319 L444–L455. 10.1152/ajplung.00252.2020 32755307 PMC7473886

[B4] Cavanagh D. (2007). Coronavirus avian infectious bronchitis virus. *Vet. Res.* 38 281–297. 17296157 10.1051/vetres:2006055

[B5] Chan C. P. Siu K. L. Chin K. T. Yuen K. Y. Zheng B. Jin D. Y. (2006). Modulation of the unfolded protein response by the severe acute respiratory syndrome coronavirus spike protein. *J. Virol.* 80 9279–9287. 10.1128/JVI.00659-06 16940539 PMC1563899

[B6] Chen Y. M. Gabler N. K. Burrough E. R. (2021). Porcine epidemic diarrhea virus infection induces endoplasmic reticulum stress and unfolded protein response in jejunal epithelial cells of weaned pigs. *Vet. Pathol.* *[Online ahead of print]* 3009858211048622. 10.1177/03009858211048622 34763602

[B7] Credle J. J. Finer-Moore J. S. Papa F. R. Stroud R. M. Walter P. (2005). On the mechanism of sensing unfolded protein in the endoplasmic reticulum. *Proc. Natl. Acad. Sci. U.S.A.* 102 18773–18784. 10.1073/pnas.0509487102 16365312 PMC1316886

[B8] Cross B. C. Bond P. J. Sadowski P. G. Jha B. K. Zak J. Goodman J. M. (2012). The molecular basis for selective inhibition of unconventional mRNA splicing by an IRE1-binding small molecule. *Proc. Natl. Acad. Sci. U.S.A.* 109 E869–E878. 10.1073/pnas.1115623109 22315414 PMC3326519

[B9] Cruz J. L. Becares M. Sola I. Oliveros J. C. Enjuanes L. Zuniga S. (2013). Alphacoronavirus protein 7 modulates host innate immune response. *J. Virol.* 87 9754–9767. 10.1128/JVI.01032-13 23824792 PMC3754097

[B10] Cruz J. L. Sola I. Becares M. Alberca B. Plana J. Enjuanes L. (2011). Coronavirus gene 7 counteracts host defenses and modulates virus virulence. *PLoS Pathog.* 7:e1002090. 10.1371/journal.ppat.1002090 PMC3111541 21695242

[B11] Datan E. Roy S. G. Germain G. Zali N. Mclean J. E. Golshan G. (2016). Dengue-induced autophagy, virus replication and protection from cell death require ER stress (PERK) pathway activation. *Cell Death Dis.* 7:e2127. 10.1038/cddis.2015.409 26938301 PMC4823927

[B12] DeDiego M. L. Nieto-Torres J. L. Jimenez-Guardeno J. M. Regla-Nava J. A. Alvarez E. Oliveros J. C. (2011). Severe acute respiratory syndrome coronavirus envelope protein regulates cell stress response and apoptosis. *PLoS Pathog.* 7:e1002315. 10.1371/journal.ppat.1002315 PMC3197621 22028656

[B13] Ding Z. An K. Xie L. Wu W. Zhang R. Wang D. (2017). Transmissible gastroenteritis virus infection induces NF-kappaB activation through RLR-mediated signaling. *Virology* 507 170–178. 10.1016/j.virol.2017.04.024 28448848 PMC7111708

[B14] Echavarria-Consuegra L. Cook G. M. Busnadiego I. Lefevre C. Keep S. Brown K. (2021). Manipulation of the unfolded protein response: a pharmacological strategy against coronavirus infection. *PLoS Pathog.* 17:e1009644. 10.1371/journal.ppat.1009644 PMC8211288 34138976

[B15] Fung T. S. Huang M. Liu D. X. (2014a). Coronavirus-induced ER stress response and its involvement in regulation of coronavirus-host interactions. *Virus Res.* 194 110–123. 10.1016/j.virusres.2014.09.016 25304691 PMC7114476

[B16] Fung T. S. Liao Y. Liu D. X. (2014b). The endoplasmic reticulum stress sensor IRE1alpha protects cells from apoptosis induced by the coronavirus infectious bronchitis virus. *J. Virol.* 88 12752–12764. 10.1128/JVI.02138-14 25142592 PMC4248946

[B17] Glimcher L. H. (2010). XBP1: the last two decades. *Ann. Rheum Dis.* 69(Suppl. 1) i67–i71. 10.1136/ard.2009.119388 19995749

[B18] Hassan I. H. Zhang M. S. Powers L. S. Shao J. Q. Baltrusaitis J. Rutkowski D. T. (2012). Influenza A viral replication is blocked by inhibition of the inositol-requiring enzyme 1 (IRE1) stress pathway. *J. Biol. Chem.* 287 4679–4689. 10.1074/jbc.M111.284695 22194594 PMC3281634

[B19] Hetz C. Papa F. R. (2017). The unfolded protein response and cell fate control. *Mol. Cell*. 69 169–181. 10.1016/j.molcel.2017.06.017 29107536

[B20] Hidalgo P. Valdes M. Gonzalez R. A. (2021). Molecular biology of coronaviruses: an overview of virus-host interactions and pathogenesis. *Bol. Med. Hosp. Infant Mex* 78 41–58. 10.24875/BMHIM.20000249 33661875

[B21] Jiang D. Niwa M. Koong A. C. (2015). Targeting the IRE1alpha-XBP1 branch of the unfolded protein response in human diseases. *Semin. Cancer Biol.* 33 48–56. 10.1016/j.semcancer.2015.04.010 25986851 PMC4523453

[B22] Krahling V. Stein D. A. Spiegel M. Weber F. Muhlberger E. (2009). Severe acute respiratory syndrome coronavirus triggers apoptosis via protein kinase R but is resistant to its antiviral activity. *J. Virol.* 83 2298–2309. 10.1128/JVI.01245-08 19109397 PMC2643707

[B23] Ksiazek T. G. Erdman D. Goldsmith C. S. Zaki S. R. Peret T. Emery S. (2003). A novel coronavirus associated with severe acute respiratory syndrome. *N. Engl. J. Med.* 348 1953–1966. 12690092 10.1056/NEJMoa030781

[B24] Landeras-Bueno S. Fernandez Y. Falcon A. Oliveros J. C. Ortin J. (2016). Chemical genomics identifies the PERK-Mediated unfolded protein stress response as a cellular target for influenza virus inhibition. *MBio* 7:e00085-16. 10.1128/mBio.00085-16 27094326 PMC4850254

[B25] Lee A. H. Iwakoshi N. N. Glimcher L. H. (2003). XBP-1 regulates a subset of endoplasmic reticulum resident chaperone genes in the unfolded protein response. *Mol. Cell Biol.* 23 7448–7459. 10.1128/MCB.23.21.7448-7459.2003 14559994 PMC207643

[B26] Liao Y. Fung T. S. Huang M. Fang S. G. Zhong Y. Liu D. X. (2013). Upregulation of CHOP/GADD153 during coronavirus infectious bronchitis virus infection modulates apoptosis by restricting activation of the extracellular signal-regulated kinase pathway. *J. Virol.* 87 8124–8134. 10.1128/JVI.00626-13 23678184 PMC3700216

[B27] Lundin A. Dijkman R. Bergstrom T. Kann N. Adamiak B. Hannoun C. (2014). Targeting membrane-bound viral RNA synthesis reveals potent inhibition of diverse coronaviruses including the middle East respiratory syndrome virus. *PLoS Pathog.* 10:e1004166. 10.1371/journal.ppat.1004166 PMC4038610 24874215

[B28] Ma Y. Wang C. Xue M. Fu F. Zhang X. Li L. (2018). The coronavirus transmissible gastroenteritis virus evades the Type I interferon response through IRE1alpha-Mediated manipulation of the MicroRNA miR-30a-5p/SOCS1/3 axis. *J. Virol.* 92 e728–e718. 10.1128/JVI.00728-18 30185587 PMC6206482

[B29] Masters P. S. (2006). The molecular biology of coronaviruses. *Adv. Virus Res.* 66 193–292. 16877062 10.1016/S0065-3527(06)66005-3 PMC7112330

[B30] Minakshi R. Padhan K. Rani M. Khan N. Ahmad F. Jameel S. (2009). The SARS Coronavirus 3a protein causes endoplasmic reticulum stress and induces ligand-independent downregulation of the type 1 interferon receptor. *PLoS One* 4:e8342. 10.1371/journal.pone.0008342 PMC2791231 20020050

[B31] Pavio N. Romano P. R. Graczyk T. M. Feinstone S. M. Taylor D. R. (2003). Protein synthesis and endoplasmic reticulum stress can be modulated by the hepatitis C virus envelope protein E2 through the eukaryotic initiation factor 2alpha kinase PERK. *J. Virol.* 77 3578–3585. 10.1128/jvi.77.6.3578-3585.2003 12610133 PMC149509

[B32] Rabouw H. H. Langereis M. A. Knaap R. C. Dalebout T. J. Canton J. Sola I. (2016). Middle east respiratory coronavirus accessory protein 4a inhibits PKR-Mediated antiviral stress responses. *PLoS Pathog.* 12:e1005982. 10.1371/journal.ppat.1005982 PMC5081173 27783669

[B33] Ron D. Walter P. (2007). Signal integration in the endoplasmic reticulum unfolded protein response. *Nat. Rev. Mol. Cell Biol.* 8 519–529. 17565364 10.1038/nrm2199

[B34] Rozpedek-Kaminska W. Siwecka N. Wawrzynkiewicz A. Wojtczak R. Pytel D. Diehl J. A. (2020). The PERK-Dependent molecular mechanisms as a novel therapeutic target for neurodegenerative diseases. *Int. J. Mol. Sci.* 21:2108. 10.3390/ijms21062108 32204380 PMC7139310

[B35] Shaban M. S. Muller C. Mayr-Buro C. Weiser H. Meier-Soelch J. Albert B. V. (2021). Multi-level inhibition of coronavirus replication by chemical ER stress. *Nat. Commun.* 12:5536. 10.1038/s41467-021-25551-1 34545074 PMC8452654

[B36] Shi C. S. Nabar N. R. Huang N. N. Kehrl J. H. (2019). SARS-coronavirus open reading Frame-8b triggers intracellular stress pathways and activates NLRP3 inflammasomes. *Cell Death Discov.* 5:101. 10.1038/s41420-019-0181-7 31231549 PMC6549181

[B37] Shuda M. Kondoh N. Imazeki N. Tanaka K. Okada T. Mori K. (2003). Activation of the ATF6, XBP1 and grp78 genes in human hepatocellular carcinoma: a possible involvement of the ER stress pathway in hepatocarcinogenesis. *J. Hepatol.* 38 605–614. 10.1016/s0168-8278(03)00029-1 12713871

[B38] Siu K. L. Chan C. P. Kok K. H. Woo P. C. Jin D. Y. (2014). Comparative analysis of the activation of unfolded protein response by spike proteins of severe acute respiratory syndrome coronavirus and human coronavirus HKU1. *Cell Biosci.* 4:3. 10.1186/2045-3701-4-3 24410900 PMC3930072

[B39] Sonkar C. Doharey P. K. Rathore A. S. Singh V. Kashyap D. Sahoo A. K. (2021). Repurposing of gastric cancer drugs against COVID-19. *Comput. Biol. Med.* 137:104826. 10.1016/j.compbiomed.2021.104826 34537409 PMC8420180

[B40] Sung S. C. Chao C. Y. Jeng K. S. Yang J. Y. Lai M. M. (2009). The 8ab protein of SARS-CoV is a luminal ER membrane-associated protein and induces the activation of ATF6. *Virology* 387 402–413. 10.1016/j.virol.2009.02.021 19304306 PMC7103415

[B41] Sureda A. Alizadeh J. Nabavi S. F. Berindan-Neagoe I. Cismaru C. A. Jeandet P. (2020). Endoplasmic reticulum as a potential therapeutic target for covid-19 infection management? *Eur. J. Pharmacol.* 882:173288. 10.1016/j.ejphar.2020.173288 32561291 PMC7297682

[B42] Tardif K. D. Mori K. Kaufman R. J. Siddiqui A. (2004). Hepatitis C virus suppresses the IRE1-XBP1 pathway of the unfolded protein response. *J. Biol. Chem.* 279 17158–17164. 10.1074/jbc.M312144200 14960590

[B43] Van Nieuwstadt A. P. Zetstra T. Boonstra J. (1989). Infection with porcine respiratory coronavirus does not fully protect pigs against intestinal transmissible gastroenteritis virus. *Vet. Rec.* 125 58–60. 10.1136/vr.125.3.58 2549676

[B44] Vela J. M. (2020). Repurposing Sigma-1 receptor ligands for COVID-19 therapy? *Front. Pharmacol.* 11:582310. 10.3389/fphar.2020.582310 PMC7751758 33364957

[B45] Versteeg G. A. Van De Nes P. S. Bredenbeek P. J. Spaan W. J. (2007). The coronavirus spike protein induces endoplasmic reticulum stress and upregulation of intracellular chemokine mRNA concentrations. *J. Virol.* 81 10981–10990. 10.1128/JVI.01033-07 17670839 PMC2045536

[B46] Wang L. Perera B. G. Hari S. B. Bhhatarai B. Backes B. J. Seeliger M. A. (2012). Divergent allosteric control of the IRE1alpha endoribonuclease using kinase inhibitors. *Nat. Chem. Biol.* 8 982–989. 10.1038/nchembio.1094 23086298 PMC3508346

[B47] Wang Y. Li J. R. Sun M. X. Ni B. Huan C. Huang L. (2014). Triggering unfolded protein response by 2-Deoxy-D-glucose inhibits porcine epidemic diarrhea virus propagation. *Antiviral Res.* 106 33–41. 10.1016/j.antiviral.2014.03.007 24681123 PMC7113873

[B48] Xue M. Fu F. Ma Y. Zhang X. Li L. Feng L. (2018). The PERK arm of the unfolded protein response negatively regulates transmissible gastroenteritis virus replication by suppressing protein translation and promoting Type I interferon production. *J. Virol.* 92 e431–e418. 10.1128/JVI.00431-18 29769338 PMC6052291

[B49] Ye Z. Wong C. K. Li P. Xie Y. (2008). A SARS-CoV protein, ORF-6, induces caspase-3 mediated, ER stress and JNK-dependent apoptosis. *Biochim. Biophys. Acta* 1780 1383–1387. 10.1016/j.bbagen.2008.07.009 18708124 PMC7115782

[B50] Yoshida H. Matsui T. Yamamoto A. Okada T. Mori K. (2001). XBP1 mRNA is induced by ATF6 and spliced by IRE1 in response to ER stress to produce a highly active transcription factor. *Cell* 107 881–891. 10.1016/s0092-8674(01)00611-0 11779464

[B51] Zarandi P. K. Zinatizadeh M. R. Zinatizadeh M. Yousefi M. H. Rezaei N. (2021). SARS-CoV-2: from the pathogenesis to potential anti-viral treatments. *Biomed. Pharmacother.* 137:111352. 10.1016/j.biopha.2021.111352 33550050 PMC7969672

[B52] Zhang J. Chen J. Shi D. Shi H. Zhang X. Liu J. (2019). Porcine deltacoronavirus enters cells via two pathways: a protease-mediated one at the cell surface and another facilitated by cathepsins in the endosome. *J. Biol. Chem.* 294 9830–9843. 10.1074/jbc.RA119.007779 31068417 PMC6597833

[B53] Zhang P. Su C. Jiang Z. Zheng C. (2017). Herpes simplex virus 1 UL41 protein suppresses the IRE1/XBP1 signal pathway of the unfolded protein response via its RNase activity. *J. Virol.* 91 e2056–e2016. 10.1128/JVI.02056-16 27928013 PMC5286897

[B54] Zhou Y. Qi B. Gu Y. Xu F. Du H. Li X. (2016). Porcine circovirus 2 deploys PERK pathway and GRP78 for its enhanced replication in PK-15 cells. *Viruses* 8:56. 10.3390/v8020056 26907328 PMC4776210

[B55] Zhu H. Zheng C. (2020). The race between host antiviral innate immunity and the immune evasion strategies of herpes simplex virus 1. *Microbiol. Mol. Biol. Rev.* 84 e99–e20. 10.1128/MMBR.00099-20 32998978 PMC7528619

[B56] Zhu L. Yang X. Mou C. Yang Q. (2017). Transmissible gastroenteritis virus does not suppress IFN-beta induction but is sensitive to IFN in IPEC-J2 cells. *Vet. Microbiol.* 199 128–134. 10.1016/j.vetmic.2016.12.031 28110779 PMC7117263

[B57] Zhu Q. C. Li S. Yuan L. X. Chen R. A. Liu D. X. Fung T. S. (2021). Induction of the proinflammatory chemokine Interleukin-8 is regulated by integrated stress response and AP-1 family proteins activated during coronavirus infection. *Int. J. Mol. Sci.* 22:5646. 10.3390/ijms22115646 34073283 PMC8198748

